# Development of O-antigen gene cluster-specific PCRs for rapid typing six epidemic serogroups of *Leptospira *in China

**DOI:** 10.1186/1471-2180-10-67

**Published:** 2010-03-03

**Authors:** Cheng-Song Cai, Yong-Zhang Zhu, Yi Zhong, Xiao-Fang Xin, Xiu-Gao Jiang, Xiao-Li Lou, Ping He, Jin-Hong Qin, Guo-Ping Zhao, Sheng-Yue Wang, Xiao-Kui Guo

**Affiliations:** 1 Department of Medical Microbiology and Parasitology, Institutes of Medical Sciences, Shanghai Jiao Tong University School of Medicine, Shanghai 200025, China; 2 Cell Biology/Key Laboratory of Synthetic Biology, Institute of Plant Physiology and Ecology, Shanghai Institutes for Biological Sciences, Chinese Academy of Sciences, Shanghai 200032, China; 3 Key Laboratory of Systems Biology, Institute of Biochemistry, Shanghai Institutes for Biological Sciences, Chinese Academy of Sciences, Shanghai 200032, China; 4 1st Bacterial Vaccine Division, National Institute for the Control of Pharmaceutical and Biological Products, Beijing 100050, China; 5 National Institute for Communicable Disease Control and Prevention, Chinese Center for Disease Control and Prevention (ICDC, China CDC), PO Box 5, Changping, Beijing 102206, China; 6 Shanghai-MOST Key Laboratory of Health and Disease Genomics, Chinese National Human Genome Center at Shanghai, Shanghai, China

## Abstract

**Background:**

*Leptospira *is the causative agent of leptospirosis. The O-antigen is the distal part of the lipopolysaccharide, which is a key component of outer membrane of Gram-negative bacteria and confers serological specificity. The epidemiology and clinical characteristics of leptospirosis are relative to the serology based taxonomic unit. Identification of *Leptospira *strains by serotyping is laborious and has several drawbacks.

**Results:**

In this study, the O-antigen gene clusters of four epidemic *Leptospira *serogroups (serogroup Canicola, Autumnalis, Grippotyphosa and Hebdomadis) in China were sequenced and all genes were predicted *in silico*. Adding published sequences of two serogroups, Icterohaemorrhagiae (strain Lai and Fiocruz L1-130) and Sejroe (strain JB197 and L550), we identified six O-antigen-specific genes for six epidemic serogroups in China. PCR assays using these genes were developed and tested on 75 reference strains and 40 clinical isolates.

**Conclusion:**

The results show that the PCR-based assays can be reliable and alternative means for rapid typing of these six serogroups of *Leptospira*.

## Background

*Leptospira*, a slender and flexuous spirochaete with tight coils, contribute to Leptospirosis [1]. The *Leptospira *genus has been divided into 20 species based on DNA-DNA hybridization studies. Pathogenic species include *L. interrogans, L. kirschneri, L. noguchii, L. borgpetersenii, L. weilii, L. santarosai, L. alexanderi *and *L. alstonii *[2-6]. *Leptospira *can also be classified into about 250 serovars based on the lipopolysaccharide (LPS) structure. Antigenically related serovars have been grouped into at least 24 serogroups [4,7].

Leptospirosis exists widely in both temperate and tropical climates and has become a serious public health threat in both developed and developing countries. Human infection results from exposure to the urine of infected animals, either directly or via contaminated soil or water[1,8]. The clinical manifestations of human leptospirosis are highly variable, ranging from mild flu-like symptoms to severe forms of infection with jaundice, pulmonary hemorrhage, multiple organ failure (mainly kidney and liver) and even death [1]. Different clinical characteristics and maintenance hosts are usually associated with certain serovars [1,8-10]. Therefore, the serology based taxonomic unit is essential for epidemiology studies, diagnosis and prevention strategies. However, *Leptospira *serotyping is performed by microscopic agglutination test (MAT) using antisera raised in rabbits against the corresponding standard references strains. This typing method is laborious and time consuming [11].

Chemical, immunochemical and ultrastructural data on LPS show that the epitope for serovar specificity is the O-antigen [1,12]. Recently, the O-antigen gene cluster of Gram-negative bacteria has been intensively studied. These genes encode proteins involved in the biosynthesis of the O-antigen and can be divided into three groups [13]. They are nucleotide sugars precursors' biosynthesis genes, glycosyltransferase genes and the O-antigen processing genes. These genes are generally found on the chromosome as an O-antigen gene (rfb) cluster. O-genotyping has been used successfully in several bacteria genus, such as *E. coli *[14], *S. enterica *[15], *S. boydii *[16], and *Y. pseudotuberculosis *[17]. Target genes of these kinds of methods are mainly the second and the third group genes that encode glycosyltransferase and O-antigen processing proteins.

DNA-based typing methods, including variable-number tandem-repeat (VNTR) typing [18-20], insertion-sequence (IS)-based typing [21,22], pulsed-filed gel electrophoresis (PFGE) [23,24], restriction fragment length polymorphism[25,26] and randomly amplified polymorphic DNA [27] have also been employed for the discrimination of serogroups of *Leptospira*. Compared with O-genotyping method, the results of these methods are not easy to analyze. Lacking of sequences of O-antigen gene clusters from various serogroups, this kind of O-genotyping has not been developed in *Leptospira*, however.

It has been confirmed that genetic variation in the O-antigen gene cluster underlies the structural variation in the O-antigen [28,29]. It has been demonstrated that O-antigen gene clusters of representative strains from different serogroups of *Leptospira *were not conservative, especially in the 5'-proximal end [30]. In this research, we sequenced the O-antigen gene cluster of four representative strains belonging to more epidemic serogroups (Canicola, Autumnalis, Grippotyphosa and Hebdomadis) in China [31]. Analyzing the O-antigen gene clusters of 8 sequenced strains (Lai, Fiocruz L1-130, JB197, L550, Gui44, Lin4, Lin6, and C401), we developed simple and practical PCR assays for six epidemic serogroups in China [32] that target serogroup-specific genes and employed to identify strains isolated from clinical samples.

## Results and Discussion

### MAT

All strains, including 75 reference strains and 40 isolated strains, were tested by MAT with standard rabbit serum. The results are shown in additional file 1 Table S1 and additional file 2 Table S2. The serology results for all reference strains are consistent with those of the National Institute for the Control of Pharmaceutical and Biological Products. Of the 40 isolated strains, 7 strains belong to serogroup Icterohaemorrhagiae,, 5 strains belong to serogroup Autumnalis, 11 strains belong to serogroup Grippotyphosa, 1 strain belongs to serogroup Hebdomadis and 5 strains belong to serogroup Sejroe. 5 isolated strains were validated by MAT as Serogroup Ballum, Australis, Javanica and Sarmin, respectively. Six strains were unable to be classified. None of strains belong to serogroup Canicola

### Development of PCR-Based Assays

We assigned functions of all ORFs by comparing homology genes. Most of predicted proteins are shown to be related to O-antigen biosynthesis except for some hypothetical proteins (see additional file 3 Table S3-6).

For typing bacteria, several different approaches have been used in *Leptospira*. Serological typing is based on strain to strain differences in the structure of lipopolysaccharide, mainly in the structure of the O-antigen. Recently, PCR-based typing methods targeting specific genes were employed for dicrimination certain serogroups of several bacteria [14-17]. These targeted genes are mainly those encoding glycosyltransferase and enzymes involved in O-antigen assembly. Among them, two highly specific genes: *wzx *(encode O-unit flippase) and *wzy *(encode O-antigen polymerase), are O-genotyping targets, usually. Previous analysis of the O-antigen of *Leptospira *showed that the biosynthesis of LPS in *Leptospira *is a Wzy-dependent pathway [12,33]. In conjunction with published data [34], our comparison of the O-antigen clusters in all 8 strains shows that the Wzy protein has a high identity among the different serogroups. Similarly, Wzx shows high similarity across other serogroups (data not shown). So we discarded these two genes as PCR assays targets.

To identify highly specific genes for PCR typing, we analyzed all predicted ORFs by the BLAST program. First, we selected genes that exhibit less than 70% amino acid similarity with their counterpart genes. Second, we compared these selected genes with draft data generated by 454 sequencing and discarded genes with more than 70% nucleotide similarity to any sequence in the draft data. *In silico *analysis of sequence of O-antigen clusters in 8 representative strains (Gui44, Lin4, Lin6, C401, JB197, L550, Fiocruz L1-130 and Lai) showed that several glycosyltransferase genes and sugar synthesis genes may be serogroup-specific. Primer pairs were designed to target these genes and PCR were performed.

**Figure 1:**
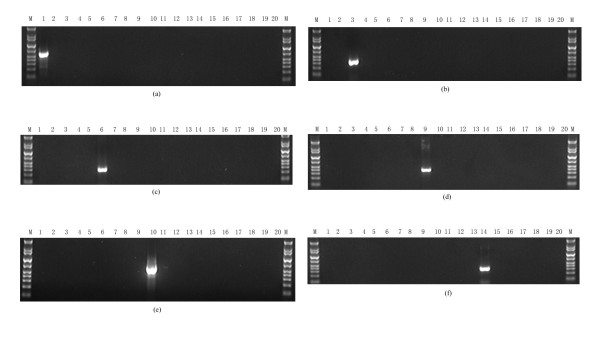
**Analysis of amplification products by electrophoresis**. Amplification products obtained by PCR of DNA pools from 18 serogroups belonging to *Leptospira *and DNA of two non-*Leptospira *strains using primer pairs ict-F/R (a), can-F/R (b), aut-F/R (c), gri-F/R (d). heb-F/R (e), sej-F/R (f). 1: Icterohaemorrhagiae; 2: Javanica; 3: Canicola; 4: Ballum; 5: Pyrogenes; 6: Autumnalis; 7: Australis; 8: Pomona; 9: Grippotyphosa; 10: Hebdomadis; 11: Bataviae; 12: Tarassovi; 13: Manhao; 14: Sejroe; 15: Mini; 16: Celledoni; 17: Ranarum; 18: Sarmin; 19: *S. enteritidis *H9812; 20: *S. aureus *N315; M: DNA marker, bands with lengths of 10 kb, 8 kb, 5 kb, 2 kb 1000 bp, 700 bp, 500 bp, 400 bp, 300 bp, 200 bp and 100 bp, respectively.

Analyzing the PCR products, we excluded primer pairs that could generate false-positive results in strains belonging to other serogroups and selected primer pairs that could discriminate as many strains belonging to the serogroups to be tested as possible. The primer pairs listed in Table 1 were our final selections. As shown in Fig. 1, DNA from strains belonging to the corresponding serogroups were able to produce PCR products of the expected size, but no PCR products were obtained from strains belonging to all other serogroups. The results of 75 reference strains are listed in additional file 1 Table S1. We also tested the specificity of six primer pairs using 40 clinically isolated strains; the results are listed in additional file 2 Table S2. All strains belonging to the six serogroups gave PCR products of the expected size with the exception of four reference strains (M49, H18, 34 and A81) belonging to the serogroup Sejroe. We speculate that the O-antigen gene clusters of these strains have been undertaken a process of recombination, where target genes may lose through recombination events. Since a few sequences of O-antigen gene clusters from *Leptospira *are available, only six serogroups of strains have been discriminated so far. There are also six strains cannot be discriminated by both MAT and O-genotyping in clinical isolates. We proposed that they are from other serogroups which beyond the field we can characterize.

**Table 1:** PCR primers targeting the specific genes

Serogroup	Target gene	Primer pairs (5'→3')	Annealing temperature(°C)	Amplicon size(bp)
Autumnalis	hypothetical protein	aut-F: TTT TGA TGG GCA TAC TGA	56	298
		aut-R: TAT GCC CTA AGT GAG TTG C		
Canicola	dTDP-4-dehydrorhamnose reductase	can-F: CAA AGG TGA TTC ACA AGG	60	341
		can-R: TCA GTG CAT TAG CCG TAT		
Grippotyphosa	glycosyltransferase	gri-F: AGA GCC GGA GGA CAG TAA	63	352
		gri-R: CGA TGG GAA ACC AAG GAT		
Hebdomadis	carbamoyl transferase	heb-F: GAT TTG ATA AGG CGA AGA	56	656
		heb-R: AAG CTC CAA TAC ATA AGG AC		
Icterohaemorrhagiae	glycosyltransferase	ict-F: TTT CAT ACG TTG CGC TTA C	57	590
		ict-R: ATA AAG TCC AGC ATC ATC CA		
Sejroe	dehydrogenase	sej-F: CGA CCG AGA TTG ACT ATG TT	60	319
		sej-R: GAA AGC AGC ATA AGT CCC		

## Conclusion

We found that six O-antigen-specific genes can be used to discriminate certain serogroups. We verified this DNA-based typing approach, which based on detecting *Leptospira *O-antigen-encoding genes, as a credible and convenient method for epidemiological research. To our knowledge, this work is the first to discriminate serogroups of *leptospira *based on the presence or absence of a PCR product.

## Methods

### Bacterial strains and culture conditions

The reference strains and clinical strains are listed in additional file 1 Table S1 and additional file 2 Table S2, respectively. All strains were grown in Ellinghausen McCullough Johnson Harris (EMJH) liquid medium at 28°C [35]. The cells were harvested at mid-log-phase by centrifugation at 12,000 × g for 15 min at 4°C.

### MAT

The MAT was performed according to the standard procedure [36] with minor modifications [37]. Live *Leptospira *cell suspensions (representing 18 serogroups) were added to serially diluted standard hyperimmune rabbit serum (from National Institute for the Control of Pharmaceutical and Biological Products) in 6-well flat-bottom microtiter plates and incubated at 37°C for 1 h. Agglutination was examined by dark-field microscopy at 100× magnification. The reported titer was calculated as the reciprocal of the highest dilution of serum that agglutinated at least 50% of the cells for each serovar used. Serogroups (serovars in parentheses) included in the antigen panel were as follows: Australis (Australis), Autumnalis (Autumnalis), Ballum (Ballum), Bataviae (Bataviae), Canicola (Canicola), Celledoni (Anhoa), Grippotyphosa (Grippotyphosa), Hebdomadis (Hebdomadis), Icterohaemorrhagiae (Lai), Javanica (Javanica), Manhao (Qingshui), Mini (Mini), Pomona (Pomona), Pyrogenes (Pyrogenes), Sejroe (Wolffi), and Tarassovi (Tarassovi).

### DNA manipulations and bioinformatic analysis

Genomic DNA was prepared with a bacterial DNA minikit (Watsonbiot, China) as previously described [38]. The genomic draft sequences of four strains (Gui44, Lin4, Lin6 and C401) were sequenced by 454 sequencing and the protocol was followed by Margulies's paper [39]. All related contigs found with a BLASTX alignment to known O-antigen genes were ordered and oriented into scaffolds with the reference strains' genomes, Lai [33], JB197, L550 [40] and Fiocruz L1-130 [41]. Sanger sequencing was performed for PCR amplicons that filled the gaps between neighboring contigs. The prediction of putative coding sequences (CDSs) and gene annotation were done by GLIMMER 3 [42] and Genemark http://opal.biology.gatech.edu/GeneMark/. ORFs were assigned functions based on a comparison with the most significant homologues in the NCBI databases and are summarized in the supplemental material.

### Specificity test of serogroup-specific PCR assay

The primers for the serogroup-specific PCR are listed in Table 1. PCR amplification was performed with 20 μl volumes containing 10× PCR buffer, 1.5 mM MgCl_2_, 100 mM deoxynucleoside triphosphates, 0.1 μM of each primer, 2.5 U Taq DNA polymerase (Takara), 50 ng template DNA and PCR-grade water. Thermal PCR conditions were as follow: initial denaturation, 95°C for 2 min; 30 cycles of 30 s at 95°C (denaturation), 30 s (annealing) at temperatures varying according to the Tm of the primer pair (annealing temperatures are listed in Table 1) and 1 min at 72°C (extension); final extension was at 72°C for 2 min. Amplification products were analyzed by electrophoresis through a 1% (wt/vol) agarose gel at 100 v for 30 min in 0.5× TBE.

The specificity of each PCR was assessed using 75 reference strains, 40 isolates and the non-*leptospira *strains of *S. enteritidis *H9812 and *S. aureus *N315.

### Nucleotide sequence accession numbers

Nucleotide sequences are available under the following accession numbers: O-antigen gene clusters of strains Gui44, Lin4, Lin6 and C401 are FJ976886, FJ976887, FJ976888 and FJ976889, respectively.

## Authors' contributions

CSC and XKG designed the research project and prepared the manuscript. CSC, YZZ and ZY carried out sequencing and data analysis. XFX and XGJ performed the strains culture and MAT. XLL, PH and JHQ performed PCR assays. GPZ and SYW participated in the design of the study and helped to draft the manuscript. All authors read and approved the final manuscript.

## Supplementary Material

Additional file 1 **Table S1: Results of reference strains discriminated with O-genotyping**. Details about 75 reference strains and O-genotyping results are included in this table. Click here for file

Additional file 2 **Table S2: Results of clinical strains discriminated with O-genotyping**. Details about 40 clinical strains and O-genotyping results are included in this table. Click here for file

Additional file 3 **Tables S3-S6**. Table S3: Putative genes in the *L. interrogans *serogroup Canicola serovar Canicola str.gui44 O-antigne gene clusterDetails about putative genes in the *L. interrogans *serogroup Canicola serovar Canicola str.gui44 O-antigne gene cluster are included in this table. Table S4: Putative genes in the *L. interrogans *serogroup Autumnalis serovar Autumnalis str.lin4 O-antigne gene clusterDetails about putative genes in the *L. interrogans *serogroup Autumnalis serovar Autumnalis str.lin4 O-antigne gene cluster are included in this table. Table S5: Putative genes in the *L. interrogans *serogroup Grippotyphosa serovar Linhai str.lin6 O-antigne gene clusterDetails about putative genes in the *L. interrogans *serogroup Grippotyphosa serovar Linhai str.lin6 O-antigne gene cluster are included in this table. Table S6: Putative genes in the *L. interrogans *serogroup Hebdomadis serovar Hebdomadis str.C401 O-antigne gene cluster. Details about putative genes in the *L. interrogans *serogroup Hebdomadis serovar Hebdomadis str.C401 O-antigne gene cluster are included in this table. Click here for file
