# Supplementary material for: Development of O-antigen gene cluster-specific PCRs for rapid typing six epidemic serogroups of Leptospira in China
Source: BMC Microbiol. 2010 Mar 3;10:67. doi: 10.1186/1471-2180-10-67 (PMC2843611; doi:10.1186/1471-2180-10-67)
Supplement: Additional file 1 — Table S1: Results of reference strains discriminated with O-genotyping. Details about 75 reference strains and O-genotyping results are included in this table. [file 1471-2180-10-67-S1.DOC]

**Additional file 1.**

**Table S1: Results of reference strains discriminated with O-genotyping**

| Strain | Serogroup*^a^* | Serovar*^b^* | Host | Year | Area | O-genotyping results | | | | | |
| --- | --- | --- | --- | --- | --- | --- | --- | --- | --- | --- | --- |
|  |  |  |  |  |  | Ict*^d^* | Can*^d^* | Aut*^d^* | Gri*^d^* | Heb*^d^* | Sej*^d^* |
| 65-9 | Australis | Australis | Horse urine | /*^c^* | Fujian | - | - | - | - | - | - |
| 507 | Australis | Rushan | Toad | 1980 | Shandong | - | - | - | - | - | - |
| Lin4 | Autumnalis | Autumnlis | Human | 1956 | Zhejiang | - | - | + | - | - | - |
| Tia1 | Autumnalis | Rachmati | Human | 1970 | Guizhou | - | - | + | - | - | - |
| L69 | Autumnalis | Bangkingnang | Human | 1964 | Yunnan | - | - | + | - | - | - |
| L56 | Autumnalis | Sumatrana | / | 1964 | Yunnan | - | - | + | - | - | - |
| L174 | Autumnalis | Mooris | Human | 1965 | Yunnan | - | - | + | - | - | - |
| A6 | Autumnalis | Nanla | Human | 1962 | Yunnan | - | - | + | - | - | - |
| GH284 | Autumnalis | Fort-bragg | Human | / | Yunnan | - | - | + | - | - | - |
| Pishu | Ballum | Ballum | / | 1964 | Sichuan | - | - | - | - | - | - |
| 1853 | Ballum | Guangdong | / | 1971 | / | - | - | - | - | - | - |
| L37 | Bataviae | Paidjan | / | 1958 | Guangxi | - | - | - | - | - | - |
| A15 | Bataviae | Bataviae | / | 1962 | / | - | - | - | - | - | - |
| lin | Canicola | Canicola | Human | 1958 | Guangdong | - | + | - | - | - | - |
| L8 | Canicola | Bindjei | Human | 1964 | Yunnan | - | + | - | - | - | - |
| A94 | Canicola | Jonsis | Human | 1970 | Yunnan | - | + | - | - | - | - |
| 7957 | Canicola | Qunjian | Rat | / | Sichuan | - | + | - | - | - | - |
| 83-194 | Canicola | Dukou | Human | / | Sichuan | - | + | - | - | - | - |
| L73 | Celledoni | Anhoa | Human | 1964 | Yunnan | - | - | - | - | - | - |
| 6712 | Celledoni | Hainan | Human | 1967 | Guangdong | - | - | - | - | - | - |
| M6906 | Celledoni | Mengdeng | Human | 1969 | Yunnan | - | - | - | - | - | - |
| 1891 | Celledoni | Whitcombi | Human | 1972 | Guangdong | - | - | - | - | - | - |
| Lin6 | Grippotyphosa | Linhai | Human | 1956 | Zhejiang | - | - | - | + | - | - |
| B8 | Grippotyphosa | Grippotyphosa | Human | 1967 | Hainan | - | - | - | + | - | - |
| 1880 | Grippotyphosa | Liangguang | Human | 1971 | Guangdong | - | - | - | + | - | - |
| P7 | Hebdomadis | Hebdomadis | Human | / | Sichuan | - | - | - | - | + | - |
| A23 | Hebdomadis | Manzhuang | Human | 1962 | Yunnan | - | - | - | - | + | - |
| M6901 | Hebdomadis | Nanding | Human | 1969 | Yunnan | - | - | - | - | + | - |
| Longnan573 | Hebdomadis | Longnan | Human | 1973 | Jiangxi | - | - | - | - | + | - |
| Lai | Icterohaemorrhagiae | Lai | Human | 1958 | Sichuan | + | - | - | - | - | - |
| 70124 | Icterohaemorrhagiae | Icterohaemorrhagiae | Human | 1969 | Sichuan | + | - | - | - | - | - |
| M37 | Icterohaemorrhagiae | Copenhageni | Human | 1960 | Yunnan | + | - | - | - | - | - |
| 1690 | Icterohaemorrhagiae | Naam | Human | 1966 | Yunnan | + | - | - | - | - | - |
| H2 | Icterohaemorrhagiae | Honghe | Human | 1959 | Yunnan | + | - | - | - | - | - |
| 81522 | Icterohaemorrhagiae | Renshou | Human | 1981 | Sichuan | + | - | - | - | - | - |
| 82224 | Icterohaemorrhagiae | Liangshan | Human | 1982 | Sichuan | + | - | - | - | - | - |
| M10 | Javanica | Javanica | Human | 1959 | Yunnan | - | - | - | - | - | - |
| A85 | Javanica | Mengla | Human | 1970 | Yunnan | - | - | - | - | - | - |
| A102 | Javanica | Mengrum | Human | 1970 | Yunnan | - | - | - | - | - | - |
| 80-27 | Javanica | Yaan | Human | 1980 | Sichuan | - | - | - | - | - | - |
| De10 | Javanica | Dehong | Human | 1981 | Yunnan | - | - | - | - | - | - |
| L82 | Javanica | Zhenkang | Rat | 1980 | Yunnan | - | - | - | - | - | - |
| S590 | Javanica | Mengma | Human | 1981 | Yunnan | - | - | - | - | - | - |
| L105 | Manhao | Cingshui | Human | 1965 | Yunnan | - | - | - | - | - | - |
| L70 | Manhao | Lushui | Human | 1964 | Yunnan | - | - | - | - | - | - |
| L60 | Manhao | Manhao | Human | 1964 | Yunnan | - | - | - | - | - | - |
| L14 | Manhao | Lincang | Human | 1977 | Yunnan | - | - | - | - | - | - |
| lichuan130 | Manhao | Lichuan | Human | 1973 | Yunnan | - | - | - | - | - | - |
| L231 | Manhao | Heyan | Human | / | Yunnan | - | - | - | - | - | - |
| A10 | Mini | Yunnan | Human | 1962 | Yunnan | - | - | - | - | - | - |
| H27 | Mini | Hekou | Human | 1964 | Yunnan | - | - | - | - | - | - |
| Nan10 | Mini | Mini | Human | / | Guangxi | - | - | - | - | - | - |
| luo | Pomona | Pomona | Human | 1960 | Fujian | - | - | - | - | - | - |
| K5 | Pomona | Kunming | Rat | 1960 | Yunnan | - | - | - | - | - | - |
| 4 | Pyrogenes | Pyrogenes | Human | 1955 | Guangdong | - | - | - | - | - | - |
| 61A | Pyrogenes | Zanoni | Human | / | Zhejiang | - | - | - | - | - | - |
| 71022 | Pyrogenes | Abramis | Human | 1971 | Zhejiang | - | - | - | - | - | - |
| S621 | Pyrogenes | Menglian | Human | 1981 | Yunnan | - | - | - | - | - | - |
| 80412 | Ranarum | Ping chang | Frog | / | Sichuan | - | - | - | - | - | - |
| S98 | Sarmin | Weaveri | Cow | 1972 | Yunnan | - | - | - | - | - | - |
| Zuang | Sejroe | Balcanica | Human | 1958 | Sichuan | - | - | - | - | - | + |
| L15 | Sejroe | Hardjo | Human | 1964 | Hainan | - | - | - | - | - | + |
| L183 | Sejroe | Wolffi | Human | 1965 | Yunnan | - | - | - | - | - | + |
| M49 | Sejroe | Medanesis | Human | 1960 | Yunnan | - | - | - | - | - | - |
| H18 | Sejroe | Haemolytica | Human | 1964 | Yunnan | - | - | - | - | - | - |
| 34 | Sejroe | Trinidad | Human | 1969 | Hainan | - | - | - | - | - | - |
| Jiao5 | Sejroe | Saxkoebing | Rat | 1980 | Hunan | - | - | - | - | - | + |
| A81 | Sejroe | Jin | Human | / | Yunnan | - | - | - | - | - | - |
| 65-52 | Tarassovi | Tarassovi | Pig | 1964 | Anhui | - | - | - | - | - | - |
| Sh71011 | Tarassovi | Guidae | Human | 1969 | Yunnan | - | - | - | - | - | - |
| A31 | Tarassovi | Banna | Human | 1962 | Yunnan | - | - | - | - | - | - |
| A82 | Tarassovi | Mengpeng | Human | 1970 | Yunnan | - | - | - | - | - | - |
| M48 | Tarassovi | Gengma | Pig | 1960 | Yunnan | - | - | - | - | - | - |
| Dong27 | Tarassovi | Moldviae | Human | 1979 | Yunnan | - | - | - | - | - | - |
| 81005 | Tarassovi | Ningxia | Pig | / | Ningxia | - | - | - | - | - | - |

*^a^* Serogroup identification was performed by MAT at National Institute for the Control of Pharmaceutical and Biological Products.

*^b^* Serovar identification was performed by cross-adsorption agglutination test at National Institute for the Control of Pharmaceutical and Biological Products.

*^c^* Data was not obtained.

*^d^* Abbreviation, Ict (Icterohaemorrhagiae), Can (Canicola), Aut (Autumnalis), Gri (Grippotyphosa), Heb (Hebdomadis), Sej (Sejroe).
