# Supplementary material for: Development of O-antigen gene cluster-specific PCRs for rapid typing six epidemic serogroups of Leptospira in China
Source: BMC Microbiol. 2010 Mar 3;10:67. doi: 10.1186/1471-2180-10-67 (PMC2843611; doi:10.1186/1471-2180-10-67)
Supplement: Additional file 2 — Table S2: Results of clinical strains discriminated with O-genotyping. Details about 40 clinical strains and O-genotyping results are included in this table. [file 1471-2180-10-67-S2.DOC]

**Additional file 2.**

**Table S2: Results of clinical strains** discriminated with O-genotyping

| Strain | Serogroup*^a^* | Serovar*^b^* | Host | Year | Area | O-genotyping results | | | | | |
| --- | --- | --- | --- | --- | --- | --- | --- | --- | --- | --- | --- |
|  |  |  |  |  |  | Ict*^e^* | Can*^e^* | Aut*^e^* | Gri*^e^* | Heb*^e^* | Sej*^e^* |
| Shuangfeng4 | Australis | ND*^c^* | /*^d^* | / | / | - | - | - | - | - | - |
| H0734 | Australis | ND | Frog | 2007 | Hunan | - | - | - | - | - | - |
| Xiangtan4 | Autumnalis | ND | Rat | 2005 | Hunan | - | - | + | - | - | - |
| 87-377 | Autumnalis | Nanla | Human | 1987 | Sichuan | - | - | + | - | - | - |
| 9701 | Autumnalis | ND | Rat | 1997 | Guangdong | - | - | + | - | - | - |
| EK7 | Autumnalis | ND | Pig | 2000 | Anhui | - | - | + | - | - | - |
| H0733 | Autumnalis | ND | Frog | 2007 | Hunan | - | - | + | - | - | - |
| Yuanjiang41 | Ballum | Ballum | / | / | / | - | - | - | - | - | - |
| Liushu4 | Grippotyphosa | Linhai | Rat | 2006 | Hunan | - | - | - | + | - | - |
| Liushu9 | Grippotyphosa | Linhai | Rat | 2006 | Hunan | - | - | - | + | - | - |
| Liushu32 | Grippotyphosa | Linhai | Rat | 2006 | Hunan | - | - | - | + | - | - |
| Liushu33 | Grippotyphosa | Linhai | Rat | 2006 | Hunan | - | - | - | + | - | - |
| Liushu35 | Grippotyphosa | Linhai | Rat | 2006 | Hunan | - | - | - | + | - | - |
| Liushu36 | Grippotyphosa | Linhai | Rat | 2006 | Hunan | - | - | - | + | - | - |
| Wa14 | Grippotyphosa | Linhai | Frog | 2006 | Hunan | - | - | - | + | - | - |
| 105 | Grippotyphosa | ND | Rat | 2001 | Guangdong | - | - | - | + | - | - |
| Shuangfeng17 | Grippotyphosa | ND | / | / | / | - | - | - | + | - | - |
| Liushu14 | Grippotyphosa | Linhai | / | / | / | - | - | - | + | - | - |
| 56653 | Grippotyphosa | Liang-guang | / | / | / | - | - | - | + | - | - |
| JC199 | Hebdomadis | ND | / | / | / | - | - | - | - | + | - |
| Shaodong43 | Icterohaemorrhagiae | Lai | Rat | 2007 | Hunan | + | - | - | - | - | - |
| Shaodong45 | Icterohaemorrhagiae | Lai | Rat | 2007 | Hunan | + | - | - | - | - | - |
| Shuangfeng24 | Icterohaemorrhagiae | ND | Rat | 2005 | / | + | - | - | - | - | - |
| Shuangfeng46 | Icterohaemorrhagiae | ND | Rat | 2005 | / | + | - | - | - | - | - |
| HN23 | Icterohaemorrhagiae | ND | Rat | 1998 | Hunan | + | - | - | - | - | - |
| HN51 | Icterohaemorrhagiae | ND | Rat | 1998 | Hunan | + | - | - | - | - | - |
| HN64 | Icterohaemorrhagiae | ND | Rat | 1998 | Hunan | + | - | - | - | - | - |
| Xiangtan7 | Javanica | ND | Rat | 2005 | Hunan | - | - | - | - | - | - |
| 56643 | Sarmin | Weaveri | / | / | / | - | - | - | - | - | - |
| Ningwa2 | Sejroe | Wolffi | Frog | 2006 | Hunan | - | - | - | - | - | + |
| Ningwa15 | Sejroe | Wolffi | Frog | 2006 | / | - | - | - | - | - | + |
| 9901 | Sejroe | ND | Human | 1999 | Guangdong | - | - | - | - | - | + |
| Yuanjiang34 | Sejroe | Saxkoebing | Rat | 2005 | Hunan | - | - | - | - | - | + |
| Yuanjiang15 | Sejroe | ND | Rat | 2006 | Hunan | - | - | - | - | - | + |
| SM6 | ND | ND | / | / | / | - | - | - | - | - | - |
| SM9 | ND | ND | / | / | / | - | - | - | - | - | - |
| G-79 | ND | ND | / | / | / | - | - | - | - | - | - |
| G-83 | ND | ND | / | / | / | - | - | - | - | - | - |
| H0743 | ND | ND | Frog | / | Hunan | - | - | - | - | - | - |
| 101 | ND | ND | / | / | / | - | - | - | - | - | - |

*^a^* Serogroup identification was performed by MAT at National Institute for Communicable Disease Control and Prevention.

*^b^* Serovar identification was performed by cross-adsorption agglutination test at National Institute for Communicable Disease Control and Prevention.

*^c^* ND, not determined.

*^d^* data was not obtained.

*^e^* Abbreviation, Ict (Icterohaemorrhagiae), Can (Canicola), Aut (Autumnalis), Gri (Grippotyphosa), Heb (Hebdomadis), Sej (Sejroe).
