# Supplementary material for: Development of O-antigen gene cluster-specific PCRs for rapid typing six epidemic serogroups of Leptospira in China
Source: BMC Microbiol. 2010 Mar 3;10:67. doi: 10.1186/1471-2180-10-67 (PMC2843611; doi:10.1186/1471-2180-10-67)
Supplement: Additional file 3 — Tables S3-S6. Table S3: Putative genes in the L. interrogans serogroup Canicola serovar Canicola str.gui44 O-antigne gene clusterDetails about putative genes in the L. interrogans serogroup Canicola serovar Canicola str.gui44 O-antigne gene cluster are included in this table. Table S4: Putative genes in the L. interrogans serogroup Autumnalis serovar Autumnalis str.lin4 O-antigne gene clusterDetails about putative genes in the L. interrogans serogroup Autumnalis serovar Autumnalis str.lin4 O-antigne gene cluster are included in this table. Table S5: Putative genes in the L. interrogans serogroup Grippotyphosa serovar Linhai str.lin6 O-antigne gene clusterDetails about putative genes in the L. interrogans serogroup Grippotyphosa serovar Linhai str.lin6 O-antigne gene cluster are included in this table. Table S6: Putative genes in the L. interrogans serogroup Hebdomadis serovar Hebdomadis str.C401 O-antigne gene cluster. Details about putative genes in the L. interrogans serogroup Hebdomadis serovar Hebdomadis str.C401 O-antigne gene cluster are included in this table. [file 1471-2180-10-67-S3.DOC]

**Additional file 3.**

Table S3: Putative genes in the *L. interrogans* serogroup Canicola serovar Canicola str.gui44 O-antigne gene cluster

| ID | Similar protein, strain | % identical aa/% similar aa (no. of aa overlap)*^a^* | Putative function of protein | Gene name |
| --- | --- | --- | --- | --- |
| gui44_rfb1 | Leptospira interrogans serovar Lai str. 56601 | 100/100(86) | MarR family transcriptional regulator |  |
| gui44_rfb2 | Leptospira interrogans serovar Lai str. 56601 | 100/100(314) | GDP-fucose synthetase | fcl |
| gui44_rfb3 | Leptospira interrogans serovar Lai str. 56601 | 99/100(365) | FAD dependent oxidoreductase |  |
| gui44_rfb4 | Leptospira interrogans serovar Lai str. 56601 | 99/99(185) | phosphoheptose isomerase |  |
| gui44_rfb5 | Leptospira interrogans serovar Lai str. 56601 | 98/99(329) | UDP-glucose 4-epimerase | galE |
| gui44_rfb6 | Leptospira interrogans serovar Lai str. 56601 | 98/100(267) | mannose-1-phosphate guanyltransferase | manC |
| gui44_rfb7 | Leptospira interrogans serovar Lai str. 56601 | 98/99(509) | ADP-heptose synthase |  |
| gui44_rfb8 | Leptospira interrogans serovar Lai str. 56601 | 99/100(330) | UDP-glucose 4-epimerase | galE |
| gui44_rfb9 | Leptospira interrogans serovar Lai str. 56601 | 34/56(359) | probable DegT/DnrJ/EryC1/StrS family protein | wecE |
| gui44_rfb10 | Leptospira interrogans serovar Lai str. 56601 | 36/60(365) | probable DegT/DnrJ/EryC1/StrS family protein | wecE |
| gui44_rfb11 | Leptospira interrogans serovar Lai str. 56601 | 38/57(543) | hypothetical protein |  |
| gui44_rfb12 | Leptospira interrogans serovar Lai str. 56601 | 68/80(260) | transketolase N-terminal section |  |
| gui44_rfb13 | Leptospira interrogans serovar Lai str. 56601 | 92/97(310) | transketolase C-terminal section |  |
| gui44_rfb14 | Leptospira interrogans serovar Lai str. 56601 | 100/100(264) | hypothetical protein |  |
| gui44_rfb15 | Leptospira interrogans serovar Lai str. 56601 | 100/100(131) | hypothetical protein |  |
| gui44_rfb16 | Leptospira interrogans serovar Lai str. 56601 | 98/99(144) | MutT/nudix family protein |  |
| gui44_rfb17 | Leptospira interrogans serovar Lai str. 56601 | 99/100(221) | methyltransferase |  |
| gui44_rfb18 | Rhodopseudomonas palustris BisA53 | 47/65(111) | methyltransferase type 11 |  |
| gui44_rfb19 | Geobacter sp. FRC-32 | 65/82(64) | Methyltransferase type 12 |  |
| gui44_rfb20 | Desulfovibrio salexigens DSM 2638 | 30/46(214) | dTDP-4-dehydrorhamnose reductase | rmlD |
| gui44_rfb21 | Leptospira borgpetersenii serovar Hardjo-bovis JB197 | 31/48(147) | methylase/methyltransferase |  |
| gui44_rfb22 | Agrobacterium tumefaciens | 25/42(244) | phytanoil-CoA alpha hydroxylase |  |
| gui44_rfb23 | Caulobacter sp. K31 | 38/59(247) | dTDP-6-deoxy-L-hexose 3-O-methyltransferase |  |
| gui44_rfb24 | Magnetococcus sp. MC-1 | 27/50(380) | methyltransferase type 12 |  |
| gui44_rfb25 | Leptospira interrogans | 90/90(63) | putative transposase protein A |  |
| gui44_rfb26 | Leptospira interrogans | 89/93(105) | putative transposase protein B |  |
| gui44_rfb27 | Leptospira interrogans serovar Lai str. 56601 | 91/97(48) | Serine acetyltransferase |  |
| gui44_rfb28 | Leptospira interrogans serovar Lai str. 56601 | 73/86(415) | hypothetical protein |  |
| gui44_rfb29 | Leptospira interrogans serovar Lai str. 56601 | 99/99(561) | ABC transporter | sunT |
| gui44_rfb30 | Leptospira interrogans serovar Lai str. 56601 | 99/100(142) | hypothetical protein |  |
| gui44_rfb31 | Leptospira interrogans serovar Lai str. 56601 | 99/99(236) | S-adenosylmethionine-dependent methyltransferase |  |
| gui44_rfb32 | Leptospira interrogans serovar Lai str. 56601 | 100/100(315) | glycosyl transferase |  |
| gui44_rfb33 | Leptospira interrogans serovar Lai str. 56601 | 100/100(557) | hypothetical protein |  |
| gui44_rfb34 | Leptospira interrogans serovar Lai str. 56601 | 97/98(228) | Probable acylneuraminate cytidylyltransferase |  |
| gui44_rfb35 | Leptospira interrogans serovar Lai str. 56601 | 99/99(330) | dTDP-glucose 4,6-dehydratase | rmlA |
| gui44_rfb36 | Leptospira interrogans serovar Lai str. 56601 | 95/97(387) | probable aminotransferase, degT family |  |
| gui44_rfb37 | Leptospira interrogans serovar Lai str. 56601 | 96/99(206) | hypothetical protein |  |
| gui44_rfb38 | Leptospira interrogans serovar Lai str. 56601 | 98/98(344) | N-acetyl neuramic acid synthetase NeuB | nnaB |
| gui44_rfb39 | Leptospira interrogans serovar Lai str. 56601 | 96/98(387) | UDP-N-acetylglucosamine-2-epimerase NeuC | nnaC |
| gui44_rfb40 | Leptospira interrogans serovar Lai str. 56601 | 99/100(351) | mannose-1-phosphate guanyltransferase | manC |
| gui44_rfb41 | Leptospira interrogans serovar Lai str. 56601 | 95/97(382) | hypothetical protein |  |
| gui44_rfb42 | Leptospira interrogans serovar Lai str. 56601 | 99/99(753) | N-acetyl neuramic acid synthetase NeuB | nnaB |
| gui44_rfb43 | Leptospira interrogans serovar Lai str. 56601 | 99/100(394) | probable DegT/DnrJ/EryC1/StrS family protein | wecE |
| gui44_rfb44 | Leptospira interrogans serovar Lai str. 56601 | 98/99(209) | Acylneuraminate cytidylyltransferase |  |
| gui44_rfb45 | Leptospira interrogans serovar Lai str. 56601 | 99/99(355) | iron alcohol dehydrogenase |  |
| gui44_rfb46 | Leptospira interrogans serovar Lai str. 56601 | 100/100(325) | molybdopterin cofactor synthesis protein A |  |
| gui44_rfb47 | Leptospira interrogans serovar Lai str. 56601 | 98/99(282) | probable methyltransferase-related protein |  |
| gui44_rfb48 | Leptospira interrogans serovar Lai str. 56601 | 99/99(602) | probable carbamoyl transferase |  |
| gui44_rfb49 | Leptospira interrogans serovar Lai str. 56601 | 95/97(586) | hypothetical protein |  |
| gui44_rfb50 | Leptospira interrogans serovar Lai str. 56601 | 94/97(219) | 6-phosphogluconolactonase | pgl |
| gui44_rfb51 | Leptospira interrogans serovar Lai str. 56601 | 98/99(198) | galactoside O-acetyltransferase |  |
| gui44_rfb52 | Leptospira interrogans serovar Lai str. 56601 | 99/99(251) | 2-dehydro-3-deoxyglucarate aldolase |  |
| gui44_rfb53 | Leptospira interrogans serovar Lai str. 56601 | 98/99(251) | 3-deoxy-manno-octulosonate cytidylyltransferase | kdsB |
| gui44_rfb54 | Leptospira interrogans serovar Lai str. 56601 | 99/99(342) | oxidoreductase |  |
| gui44_rfb55 | Leptospira interrogans serovar Lai str. 56601 | 96/98(257) | inositol monophophatase family protein |  |
| gui44_rfb56 | Leptospira interrogans serovar Lai str. 56601 | 99/99(246) | Ribosomal protein S27e |  |
| gui44_rfb57 | Leptospira interrogans serovar Lai str. 56601 | 100/100(332) | phosphoglycerate dehydrogenase |  |
| gui44_rfb58 | Leptospira interrogans serovar Lai str. 56601 | 99/99(629) | hypothetical protein |  |
| gui44_rfb59 | Leptospira interrogans serovar Lai str. 56601 | 100/100(255) | Glucose-1-phosphate cytidylyltransferase | ddhA |
| gui44_rfb60 | Leptospira interrogans serovar Lai str. 56601 | 99/99(363) | CDP-glucose 4,6-dehydratase | ddhB |
| gui44_rfb61 | Leptospira interrogans serovar Lai str. 56601 | 99/99(150) | dTDP-4-dehydrorhamnose 3,5-epimerase and related enzyme |  |
| gui44_rfb62 | Leptospira interrogans serovar Lai str. 56601 | 100/100(317) | UDP-glucose 4-epimerase | galE |
| gui44_rfb63 | Leptospira interrogans serovar Lai str. 56601 | 98/99(298) | glycosyl transferase |  |
| gui44_rfb64 | Leptospira interrogans serovar Lai str. 56601 | 93/94(135) | hypothetical protein |  |
| gui44_rfb65 | Leptospira interrogans serovar Lai str. 56601 | 94/96(287) | Polysaccharide deacetylase-like protein |  |
| gui44_rfb66 | Leptospira interrogans serovar Lai str. 56601 | 98/99(704) | FemAB family protein |  |
| gui44_rfb67 | Leptospira interrogans serovar Lai str. 56601 | 100/100(673) | lipoprotein |  |
| gui44_rfb68 | Leptospira interrogans serovar Lai str. 56601 | 98/99(125) | hypothetical protein |  |
| gui44_rfb69 | Leptospira interrogans serovar Lai str. 56601 | 48/66(377) | glycosyl transferase |  |
| gui44_rfb70 | Leptospira interrogans serovar Lai str. 56601 | 99/99(344) | UDP-glucose 4-epimerase | galE |
| gui44_rfb71 | Leptospira interrogans serovar Lai str. 56601 | 99/99(366) | epimerase |  |
| gui44_rfb72 | Leptospira interrogans serovar Lai str. 56601 | 99/99(376) | UDP-N-acetylglucosamine 2-epimerase | gne |
| gui44_rfb73 | Leptospira interrogans serovar Lai str. 56601 | 99/99(395) | glycosyl transferase |  |
| gui44_rfb74 | Leptospira interrogans serovar Lai str. 56601 | 99/100(421) | colanic biosynthesis UDP-glucose lipid carrier transferase | wcaJ |
| gui44_rfb75 | Leptospira interrogans serovar Lai str. 56601 | 98/99(422) | hypothetical protein |  |
| gui44_rfb76 | Leptospira interrogans serovar Lai str. 56601 | 99/99(447) | polysaccharide biosynthesis protein |  |
| gui44_rfb77 | Leptospira borgpetersenii serovar Hardjo-bovis L550 | 87/95(300) | putative glycosyltransferase |  |
| gui44_rfb78 | Leptospira interrogans serovar Copenhageni str. Fiocruz L1-130 | 99/100(183) | hypothetical protein |  |
| gui44_rfb79 | Leptospira borgpetersenii serovar Hardjo-bovis L550 | 99/100(325) | glycosyltransferase |  |
| gui44_rfb80 | Leptospira interrogans serovar Lai str. 56601 | 90/97(138) | sugar isomerase |  |
| gui44_rfb81 | Leptospira interrogans serovar Copenhageni str. Fiocruz L1-130 | 99/99(363) | DegT/DnrJ/EryC1/StrS family protein | wecE |
| gui44_rfb82 | Leptospira interrogans serovar Lai str. 56601 | 99/100(228) | glycosyltransferase |  |
| gui44_rfb83 | Leptospira interrogans serovar Copenhageni str. Fiocruz L1-130 | 99/100(273) | glycosyl transferase |  |
| gui44_rfb84 | Leptospira interrogans serovar Lai str. 56601 | 99/99(582) | glycosyl transferase | rmlC |
| gui44_rfb85 | Leptospira interrogans serovar Lai str. 56601 | 100/100(186) | dTDP-4-dehydrorhamnose 3,5-epimerase | rmlD |
| gui44_rfb86 | Leptospira interrogans serovar Lai str. 56601 | 97/99(306) | dTDP-4-dehydrorhamnose reductase | rmlB |
| gui44_rfb87 | Leptospira interrogans serovar Copenhageni str. Fiocruz L1-130 | 99/99(336) | dTDP-glucose 4,6-dehydratase | rmlA |
| gui44_rfb88 | Leptospira interrogans serovar Lai str. 56601 | 100/100(294) | glucose-1-phosphate thymidylyltransferase |  |
| gui44_rfb89 | Leptospira interrogans serovar Lai str. 56601 | 99/100(324) | glycosyl transferase |  |
| gui44_rfb90 | Leptospira interrogans serovar Lai str. 56601 | 99/99(303) | dTDP-rhamnosyl transferase rfbF |  |
| gui44_rfb91 | Leptospira interrogans serovar Lai str. 56601 | 98/99(282) | glycosyl transferase |  |
| gui44_rfb92 | Leptospira interrogans serovar Copenhageni str. Fiocruz L1-130 | 99/100(464) | Sodium:sulfate symporter | citT |

*^a^* aa, amino acid.

Table S4: Putative genes in the *L. interrogans* serogroup Autumnalis serovar Autumnalis str.lin4 O-antigne gene cluster

| ID | Similar protein, strain | % identical aa/% similar aa (no. of aa overlap)*^a^* | Putative function of protein | Gene name |
| --- | --- | --- | --- | --- |
| lin4_rfb1 | Leptospira interrogans serovar Lai str. 56601 | 100/100(86) | MarR family transcriptional regulator |  |
| lin4_rfb2 | Leptospira interrogans serovar Lai str. 56601 | 100/100(314) | GDP-fucose synthetase | fcl |
| lin4_rfb3 | Leptospira interrogans serovar Lai str. 56601 | 99/100(365) | FAD dependent oxidoreductase |  |
| lin4_rfb4 | Leptospira interrogans serovar Lai str. 56601 | 100/100(185) | Phosphoheptose isomerase | gmhA |
| lin4_rfb5 | Leptospira interrogans serovar Lai str. 56601 | 99/99(329) | UDP-glucose 4-epimerase | galE |
| lin4_rfb6 | Leptospira interrogans serovar Lai str. 56601 | 99/100(233) | mannose-1-phosphate guanyltransferase | manC |
| lin4_rfb7 | Leptospira interrogans serovar Lai str. 56601 | 98/99(527) | ADP-heptose synthetase | rfaE |
| lin4_rfb8 | Leptospira interrogans serovar Lai str. 56601 | 99/100(330) | UDP-glucose 4-epimerase | galE |
| lin4_rfb9 | Leptospira interrogans serovar Lai str. 56601 | 34/56(359) | probable DegT/DnrJ/EryC1/StrS family protein | wecE |
| lin4_rfb10 | Leptospira interrogans serovar Lai str. 56601 | 36/60(365) | probable DegT/DnrJ/EryC1/StrS family protein | wecE |
| lin4_rfb11 | Leptospira interrogans serovar Lai str. 56601 | 38/58(547) | hypothetical protein |  |
| lin4_rfb12 | Leptospira interrogans serovar Lai str. 56601 | 67/79(264) | transketolase N-terminal section |  |
| lin4_rfb13 | Leptospira interrogans serovar Lai str. 56601 | 92/97(310) | transketolase C-terminal section |  |
| lin4_rfb14 | Leptospira interrogans serovar Lai str. 56601 | 99/99(264) | hypothetical protein |  |
| lin4_rfb15 | Leptospira interrogans serovar Lai str. 56601 | 100/100(131) | hypothetical protein |  |
| lin4_rfb16 | Leptospira interrogans serovar Lai str. 56601 | 98/99(144) | MutT/nudix family protein |  |
| lin4_rfb17 | Leptospira interrogans serovar Lai str. 56601 | 99/99(221) | methyltransferase |  |
| lin4_rfb18 | Leptospira interrogans serovar Lai str. 56601 | 100/100(175) | hypothetical protein |  |
| lin4_rfb19 | Leptospira interrogans serovar Lai str. 56601 | 99/99(405) | D-mycarose 3-C-methyltransferase |  |
| lin4_rfb20 | Leptospira interrogans serovar Lai str. 56601 | 98/99(358) | perosamine synthetase |  |
| lin4_rfb21 | Leptospira interrogans serovar Lai str. 56601 | 97/99(308) | methionyl-tRNA(fmet) n-formyltransferase |  |
| lin4_rfb22 | Leptospira interrogans serovar Lai str. 56601 | 31/52(376) | hypothetical protein |  |
| lin4_rfb23 | Leptospira interrogans serovar Lai str. 56601 | 29/50(199) | hypothetical protein |  |
| lin4_rfb24 | Leptospira interrogans serovar Lai str. 56601 | 35/54(375) | Probable transport ATP-binding protein msbA | sunT |
| lin4_rfb25 | Fusobacterium nucleatum subsp.nucleatum ATCC 25586 | 34/56(269) | dTDP-4-dehydrorhamnose reductase | rmlD |
| lin4_rfb26 | Dechloromonas aromatica RCB | 67/79(392) | probable DegT/DnrJ/EryC1/StrS family protein | wecE |
| lin4_rfb27 | Dechloromonas aromatica RCB | 46/64(288) | aldo/keto reductase |  |
| lin4_rfb28 | Methanocaldococcus jannaschii DSM 2661 | 32/52(296) | spore coat polysaccharide biosynthesis protein E | spsE |
| lin4_rfb29 | Methanocaldococcus jannaschii DSM 2661 | 28/47(233) | spore coat polysaccharide biosynthesis protein F | spsF |
| lin4_rfb30 | Sulfurimonas denitrificans DSM 1251 | 47/64(428) | glutamate-1-semialdehyde 2,1-aminomutase |  |
| lin4_rfb31 | Sulfurimonas denitrificans DSM 1251 | 52/68(261) | Nitrilase/cyanide hydratase and apolipoprotein N-acyltransferase |  |
| lin4_rfb32 | Leptospira biflexa serovar Patoc strain 'Patoc 1 (Paris)' | 46/64(285) | putative glycosyltransferase |  |
| lin4_rfb33 | Leptospira biflexa serovar Patoc strain 'Patoc 1 (Ames)' | 40/59(129) | acetyltransferase |  |
| lin4_rfb34 | Geobacillus sp. Y412MC61 | 70/84(334) | N-acylneuraminate-9-phosphate synthase |  |
| lin4_rfb35 | Leptospira interrogans serovar Lai str. 56601 | 94/97(207) | transposase |  |
| lin4_rfb36 | No homology gene was found | / | hypothetical protein |  |
| lin4_rfb37 | Campylobacter jejuni subsp.jejuni CG8486 | 23/40(300) | DegT/DnrJ/EryC1/StrS aminotransferase family protein | wecE |
| lin4_rfb38 | No homology gene was found | / | hypothetical protein |  |
| lin4_rfb39 | Shewanella denitrificans OS217 | 29/50(453) | FAD dependent oxidoreductase |  |
| lin4_rfb40 | No homology gene was found | / | hypothetical protein |  |
| lin4_rfb41 | Acaryochloris marina MBIC11017 | 49/68(258) | 2-dehydro-3-deoxyphosphooctonate aldolase | garL |
| lin4_rfb42 | Desulfobacterium autotrophicum HRM2 | 36/56(121) | KdsD |  |
| lin4_rfb43 | No homology gene was found | / | hypothetical protein |  |
| lin4_rfb44 | Campylobacter coli RM2228 | 36/56(252) | 2,4-dihydroxyhept-2-ene-1,7-dioic acid aldolase |  |
| lin4_rfb45 | Opitutaceae bacterium TAV2 | 51/67(259) | short-chain dehydrogenase/reductase SDR |  |
| lin4_rfb46 | No homology gene was found | / | hypothetical protein |  |
| lin4_rfb47 | Cyanothece sp. PCC 8802 | 25/44(247) | Methyltransferase type 12 |  |
| lin4_rfb48 | No homology gene was found | / | hypothetical protein |  |
| lin4_rfb49 | Lactobacillus reuteri DSM 20016 | 37/55(254) | short-chain dehydrogenase/reductase SDR |  |
| lin4_rfb50 | Leptospira interrogans serovar Copenhageni str. Fiocruz L1-130 | 34/54(253) | 3-deoxy-manno-octulosonate cytidylyltransferase | kdsB |
| lin4_rfb51 | No homology gene was found | / | hypothetical protein |  |
| lin4_rfb52 | No homology gene was found | / | hypothetical protein |  |
| lin4_rfb53 | Parabacteroides distasonis ATCC 8503 | 39/53(71) | glycosyl transferase family protein |  |
| lin4_rfb54 | No homology gene was found | / | hypothetical protein |  |
| lin4_rfb55 | Methylobacterium nodulans ORS 2060 | 45/71(124) | Methyltransferase type 12 |  |
| lin4_rfb56 | No homology gene was found | / | hypothetical protein |  |
| lin4_rfb57 | Leptospira interrogans | 88/92(144) | putative transposase protein A |  |
| lin4_rfb58 | Leptospira interrogans | 86/90(119) | putative transposase protein B |  |
| lin4_rfb59 | No homology gene was found | / | hypothetical protein |  |
| lin4_rfb60 | Leptospira interrogans serovar Lai str. 56601 | 48/66(377) | glycosyl transferase |  |
| lin4_rfb61 | Leptospira interrogans serovar Copenhageni str. Fiocruz L1-130 | 100/100(344) | UDP-glucose 4-epimerase | galE |
| lin4_rfb62 | Leptospira interrogans serovar Copenhageni str. Fiocruz L1-130 | 100/100(366) | nucleoside-diphosphate-sugar epimerase |  |
| lin4_rfb63 | Leptospira interrogans serovar Lai str. 56601 | 99/99(376) | UDP-N-acetylglucosamine 2-epimerase | wecB |
| lin4_rfb64 | Leptospira interrogans serovar Lai str. 56601 | 99/99(395) | glycosyl transferase |  |
| lin4_rfb65 | Leptospira interrogans serovar Lai str. 56601 | 100/100(421) | colanic biosynthesis UDP-glucose lipid carrier transferase | wcaJ |
| lin4_rfb66 | Leptospira interrogans serovar Lai str. 56601 | 99/99(422) | hypothetical protein |  |
| lin4_rfb67 | Leptospira interrogans serovar Lai str. 56601 | 99/100(447) | polysaccharide biosynthesis protein |  |
| lin4_rfb68 | Leptospira borgpetersenii serovar Hardjo-bovis L550 | 87/95(300) | putative glycosyltransferase |  |
| lin4_rfb69 | Leptospira interrogans serovar Lai str. 56601 | 99/99(297) | hypothetical protein |  |
| lin4_rfb70 | Leptospira interrogans serovar Lai str. 56601 | 99/99(340) | hypothetical protein |  |
| lin4_rfb71 | Leptospira interrogans serovar Lai str. 56601 | 99/100(139) | hypothetical protein |  |
| lin4_rfb72 | Leptospira interrogans serovar Lai str. 56601 | 100/100(363) | DegT/DnrJ/EryC1/StrS family protein | wecE |
| lin4_rfb73 | Leptospira interrogans serovar Lai str. 56601 | 99/99(265) | glycosyl transferase |  |
| lin4_rfb74 | Leptospira interrogans serovar Lai str. 56601 | 100/100(273) | glycosyl transferase |  |
| lin4_rfb75 | Leptospira interrogans serovar Lai str. 56601 | 99/100(578) | glycosyl transferase |  |
| lin4_rfb76 | Leptospira interrogans serovar Lai str. 56601 | 100/100(186) | dTDP-4-dehydrorhamnose 3,5-epimerase | rmlC |
| lin4_rfb77 | Leptospira interrogans serovar Lai str. 56601 | 99/100(306) | dTDP-4-dehydrorhamnose reductase | rmlD |
| lin4_rfb78 | Leptospira interrogans serovar Lai str. 56601 | 100/100(336) | dTDP-glucose 4,6-dehydratase | rmlB |
| lin4_rfb79 | Leptospira interrogans serovar Lai str. 56601 | 100/100(294) | Glucose-1-phosphate thymidylyltransferase | rmlA |
| lin4_rfb80 | Leptospira interrogans serovar Lai str. 56601 | 100/100(324) | glycosyl transferase |  |
| lin4_rfb81 | Leptospira interrogans serovar Lai str. 56601 | 99/100(303) | dTDP-rhamnosyl transferase rfbF |  |
| lin4_rfb82 | Leptospira interrogans serovar Lai str. 56601 | 99/100(282) | glycosyl transferase |  |
| lin4_rfb83 | Leptospira interrogans serovar Lai str. 56601 | 99/99(464) | Sodium:sulfate symporter | citT |

*^a^* aa, amino acid.

Table S5: Putative genes in the *L. interrogans* serogroup Grippotyphosa serovar Linhai str.lin6 O-antigne gene cluster

| ID | Similar protein, strain | % identical aa/% similar aa (no. of aa overlap)*^a^* | Putative function of protein | Gene name |
| --- | --- | --- | --- | --- |
| lin6_rfb1 | Leptospira interrogans serovar Lai str. 56601 | 100/100(106) | Transcriptional regulator, marR family |  |
| lin6_rfb2 | Leptospira interrogans serovar Lai str. 56601 | 100/100(314) | GDP-fucose synthetase | fcl |
| lin6_rfb3 | Leptospira interrogans serovar Lai str. 56601 | 99/100(365) | FAD dependent oxidoreductase |  |
| lin6_rfb4 | Leptospira interrogans serovar Lai str. 56601 | 100/100(185) | Phosphoheptose isomerase | gmhA |
| lin6_rfb5 | Leptospira interrogans serovar Lai str. 56601 | 99/100(329) | UDP-glucose 4-epimerase | galE |
| lin6_rfb6 | Leptospira interrogans serovar Lai str. 56601 | 99/99(267) | mannose-1-phosphate guanyltransferase | manC |
| lin6_rfb7 | Leptospira interrogans serovar Lai str. 56601 | 98/99(509) | ADP-heptose synthase | rfaE |
| lin6_rfb8 | Leptospira interrogans serovar Lai str. 56601 | 99/100(330) | UDP-glucose 4-epimerase | galE |
| lin6_rfb9 | Leptospira interrogans serovar Lai str. 56601 | 34/56(359) | probable DegT/DnrJ/EryC1/StrS family protein | wecE |
| lin6_rfb10 | Leptospira interrogans serovar Lai str. 56601 | 36/60(365) | probable DegT/DnrJ/EryC1/StrS family protein | wecE |
| lin6_rfb11 | Leptospira interrogans serovar Lai str. 56601 | 38/57(543) | hypothetical protein |  |
| lin6_rfb12 | Leptospira interrogans serovar Lai str. 56601 | 68/80(260) | transketolase N-terminal section |  |
| lin6_rfb13 | Leptospira interrogans serovar Lai str. 56601 | 92/97(310) | transketolase C-terminal section |  |
| lin6_rfb14 | Leptospira interrogans serovar Lai str. 56601 | 99/99(264) | hypothetical protein |  |
| lin6_rfb15 | Leptospira interrogans serovar Lai str. 56601 | 100/100(131) | hypothetical protein |  |
| lin6_rfb16 | Leptospira interrogans serovar Lai str. 56601 | 98/99(144) | MutT/nudix family protein |  |
| lin6_rfb17 | Leptospira interrogans serovar Lai str. 56601 | 99/100(221) | methyltransferase |  |
| lin6_rfb18 | Leptospira interrogans serovar Lai str. 56601 | 100/100(175) | hypothetical protein |  |
| lin6_rfb19 | Leptospira interrogans serovar Lai str. 56601 | 98/99(399) | D-mycarose 3-C-methyltransferase |  |
| lin6_rfb20 | Chlorobium ferrooxidans DSM 13031 | 34/53(239) | Short-chain dehydrogenase/reductase SDR |  |
| lin6_rfb21 | Magnetococcus sp. MC-1 | 26/47(378) | methyltransferase type 12 |  |
| lin6_rfb22 | Leptospira interrogans serovar Lai str. 56601 | 30/51(397) | hypothetical protein |  |
| lin6_rfb23 | Leptospira interrogans serovar Lai str. 56601 | 29/51(200) | hypothetical protein |  |
| lin6_rfb24 | Leptospira interrogans serovar Lai str. 56601 | 30/49(520) | Probable transport ATP-binding protein msbA | sunT |
| lin6_rfb25 | Leptospira interrogans serovar Lai str. 56601 | 28/43(297) | dTDP-4-dehydrorhamnose reductase | rmlD |
| lin6_rfb26 | Leptospira interrogans serovar Lai str. 56601 | 32/52(390) | probable DegT/DnrJ/EryC3/StrS family protein | wecE |
| lin6_rfb27 | Dechloromonas aromatica RCB | 46/64(288) | aldo/keto reductase |  |
| lin6_rfb28 | Leptospira interrogans serovar Lai str. 56601 | 27/44(339) | N-acetyl neuramic acid synthetase NeuB | nnaB |
| lin6_rfb29 | Leptospira interrogans serovar Lai str. 56601 | 28/49(234) | spore coat polysaccharide biosynthesis protein spsF-like protein |  |
| lin6_rfb30 | Sulfurimonas denitrificans DSM 1251 | 47/64(428) | glutamate-1-semialdehyde 2,1-aminomutase |  |
| lin6_rfb31 | Leptospira interrogans serovar Lai str. 56601 | 33/52(259) | probable carbon-nitrogen hydrolase |  |
| lin6_rfb32 | Leptospira biflexa serovar Patoc strain 'Patoc 1 | 48/65(275) | putative glycosyltransferase |  |
| lin6_rfb33 | Leptospira interrogans serovar Lai str. 56601 | 35/49(127) | methionyl-tRNA formyltransferase |  |
| lin6_rfb34 | Leptospira interrogans serovar Lai str. 56601 | 34/57(324) | N-acetyl neuramic acid synthetase NeuB | nnaB |
| lin6_rfb35 | Leptospira biflexa serovar Patoc strain 'Patoc 1 | 20/40(187) | glycosyltransferase |  |
| lin6_rfb36 | marine gamma proteobacterium HTCC2080 | 55/72(236) | methyltransferase domain protein |  |
| lin6_rfb37 | No homology gene was found | / | hypothetical protein |  |
| lin6_rfb38 | Leptospira interrogans serovar Lai str. 56601 | 40/65(253) | 2-dehydro-3-deoxyphosphooctonate aldolase | kdsA |
| lin6_rfb39 | Desulfobacterium autotrophicum HRM2 | 36/55(112) | KdsD |  |
| lin6_rfb40 | Leptospira interrogans serovar Lai str. 56601 | 34/53(236) | 2-dehydro-3-deoxyglucarate aldolase | garL |
| lin6_rfb41 | Leptospira interrogans serovar Lai str. 56601 | 32/49(249) | 3-oxoacyl-[acyl-carrier protein] reductase | fabG |
| lin6_rfb42 | Leptospira interrogans serovar Lai str. 56601 | 40/60(190) | Galactoside O-acetyltransferase | wbbJ |
| lin6_rfb43 | Cyanothece sp. PCC 8802 | 24/45(260) | Methyltransferase type 12 |  |
| lin6_rfb44 | No homology gene was found | / | hypothetical protein |  |
| lin6_rfb45 | Leptospira interrogans serovar Lai str. 56601 | 30/50(244) | 3-oxoacyl-[acyl-carrier protein] reductase | fabG |
| lin6_rfb46 | Leptospira interrogans serovar Lai str. 56601 | 33/54(254) | 3-deoxy-manno-octulosonate cytidylyltransferase | kdsB |
| lin6_rfb47 | No homology gene was found | / | hypothetical protein |  |
| lin6_rfb48 | No homology gene was found | / | hypothetical protein |  |
| lin6_rfb49 | Synechococcus sp. WH 7805 | 50/69(153) | phosphatase kdsC |  |
| lin6_rfb50 | No homology gene was found | / | hypothetical protein |  |
| lin6_rfb51 | No homology gene was found | / | hypothetical protein |  |
| lin6_rfb52 | Methylobacterium nodulans ORS 2060 | 41/64(236) | Methyltransferase type 12 |  |
| lin6_rfb53 | Leptospira interrogans serovar Lai str. 56601 | 65/78(674) | lipoprotein |  |
| lin6_rfb54 | Leptospira interrogans serovar Lai str. 56601 | 88/92(431) | hypothetical protein |  |
| lin6_rfb55 | Leptospira interrogans serovar Lai str. 56601 | 48/66(377) | glycosyl transferase |  |
| lin6_rfb56 | Leptospira interrogans serovar Lai str. 56601 | 99/99(344) | UDP-glucose 4-epimerase | galE |
| lin6_rfb57 | Leptospira interrogans serovar Lai str. 56601 | 99/99(366) | epimerase |  |
| lin6_rfb58 | Leptospira interrogans serovar Lai str. 56601 | 99/99(376) | UDP-N-acetylglucosamine 2-epimerase | wecB |
| lin6_rfb59 | Leptospira interrogans serovar Lai str. 56601 | 99/99(395) | glycosyl transferase |  |
| lin6_rfb60 | Leptospira interrogans serovar Lai str. 56601 | 99/100(421) | colanic biosynthesis UDP-glucose lipid carrier transferase | wcaJ |
| lin6_rfb61 | Leptospira interrogans serovar Lai str. 56601 | 100/100(414) | hypothetical protein |  |
| lin6_rfb62 | Leptospira interrogans serovar Lai str. 56601 | 99/99(429) | polysaccharide biosynthesis protein |  |
| lin6_rfb63 | Leptospira borgpetersenii serovar Hardjo-bovis L550 | 87/95(300) | putative glycosyltransferase |  |
| lin6_rfb64 | Leptospira interrogans serovar Lai str. 56601 | 100/100(183) | hypothetical protein |  |
| lin6_rfb65 | Leptospira interrogans serovar Lai str. 56601 | 99/99(301) | hypothetical protein |  |
| lin6_rfb66 | Leptospira interrogans serovar Lai str. 56601 | 100/100(139) | hypothetical protein |  |
| lin6_rfb67 | Leptospira interrogans serovar Lai str. 56601 | 100/100(363) | probable DegT/DnrJ/EryC1/StrS family protein | wecE |
| lin6_rfb68 | Leptospira interrogans serovar Lai str. 56601 | 99/99(265) | glycosyl transferase |  |
| lin6_rfb69 | Leptospira interrogans serovar Lai str. 56601 | 100/100(273) | glycosyl transferase |  |
| lin6_rfb70 | Leptospira interrogans serovar Lai str. 56601 | 99/100(582) | glycosyl transferase |  |
| lin6_rfb71 | Leptospira interrogans serovar Lai str. 56601 | 100/100(186) | dTDP-4-dehydrorhamnose 3,5-epimerase | rmlC |
| lin6_rfb72 | Leptospira interrogans serovar Lai str. 56601 | 97/99(306) | dTDP-4-dehydrorhamnose reductase | rmlD |
| lin6_rfb73 | Leptospira interrogans serovar Lai str. 56601 | 99/99(349) | dTDP-glucose 4,6-dehydratase | rmlB |
| lin6_rfb74 | Leptospira interrogans serovar Lai str. 56601 | 100/100(294) | Glucose-1-phosphate thymidylyltransferase | rmlA |
| lin6_rfb75 | Leptospira interrogans serovar Lai str. 56601 | 100/100(324) | glycosyl transferase |  |
| lin6_rfb76 | Leptospira interrogans serovar Lai str. 56601 | 100/100(303) | dTDP-rhamnosyl transferase rfbF |  |
| lin6_rfb77 | Leptospira interrogans serovar Lai str. 56601 | 99/100(282) | glycosyl transferase |  |
| lin6_rfb78 | Leptospira interrogans serovar Lai str. 56601 | 99/99(464) | Sodium:sulfate symporter | citT |

*^a^* aa, amino acid.

Table S6: Putative genes in the *L. interrogans* serogroup Hebdomadis serovar Hebdomadis str.C401 O-antigne gene cluster

| ID | Similar protein, strain | % identical aa/% similar aa (no. of aa overlap)*^a^* | Putative function of protein | Gene name |
| --- | --- | --- | --- | --- |
| C401_rfb1 | Leptospira interrogans serovar Lai str. 56601 | 100/100(86) | MarR family transcriptional regulator |  |
| C401_rfb2 | Leptospira interrogans serovar Lai str. 56601 | 99/99(310) | GDP-fucose synthetase | fcl |
| C401_rfb3 | Leptospira borgpetersenii serovar Hardjo-bovis JB197 | 97/98(361) | alcohol dehyodrogenase |  |
| C401_rfb4 | Leptospira borgpetersenii serovar Hardjo-bovis L550 | 95/98(219) | glycosyltransferase |  |
| C401_rfb5 | Leptospira borgpetersenii serovar Hardjo-bovis L550 | 97/99(354) | N-acetylneuraminic acid (sialic acid) synthetase | neuB |
| C401_rfb6 | Leptospira borgpetersenii serovar Hardjo-bovis L550 | 93/97(229) | cytidylyltransferase |  |
| C401_rfb7 | Dvul_0366, Desulfovibrio vulgaris subsp. vulgaris DP4 | 32/50(128) | acylneuraminate cytidylyltransferase |  |
| C401_rfb8 | Leptospira borgpetersenii serovar Hardjo-bovis L550 | 97/99(599) | carbamoyl transferase |  |
| C401_rfb9 | Leptospira borgpetersenii serovar Hardjo-bovis L550 | 97/99(387) | pyridoxal phosphate-dependent aminotransferase | nifS |
| C401_rfb10 | Leptospira borgpetersenii serovar Hardjo-bovis L550 | 97/99(583) | carbamoyl transferase |  |
| C401_rfb11 | Leptospira borgpetersenii serovar Hardjo-bovis L550 | 93/97(537) | dehydrogenase |  |
| C401_rfb12 | Leptospira borgpetersenii serovar Hardjo-bovis L550 | 93/96(417) | pyridoxal-phosphate-dependent aminotransferase | nifS |
| C401_rfb13 | Leptospira borgpetersenii serovar Hardjo-bovis L550 | 87/95(345) | hypothetical protein |  |
| C401_rfb14 | Leptospira borgpetersenii serovar Hardjo-bovis L550 | 93/97(180) | acetyltransferase |  |
| C401_rfb15 | Leptospira interrogans serovar Lai str. 56601 | 44/58(606) | carbamoyl transferase |  |
| C401_rfb16 | Leptospira borgpetersenii serovar Hardjo-bovis L550 | 39/63(216) | methyltransferase |  |
| C401_rfb17 | Spiroplasma apis | 25/41(212) | Chromosomal replication initiator protein | dnaA |
| C401_rfb18 | Desulfococcus oleovorans Hxd3 | 44/68(206) | transposase IS4 family protein |  |
| C401_rfb19 | Ochrobactrum anthropi ATCC 49188 | 42/65(82) | transposase IS4 family protein |  |
| C401_rfb20 | Leptospira borgpetersenii serovar Hardjo-bovis L550 | 85/92(449) | aminopeptidase |  |
| C401_rfb21 | Leptospira borgpetersenii serovar Hardjo-bovis L550 | 88/95(242) | cytidylyltransferase |  |
| C401_rfb22 | Leptospira borgpetersenii serovar Hardjo-bovis JB197 | 90/94(453) | hypothetical protein |  |
| C401_rfb23 | Leptospira borgpetersenii serovar Hardjo-bovis L550 | 96/98(255) | Short chain dehydrogenase |  |
| C401_rfb24 | Leptospira borgpetersenii serovar Hardjo-bovis L550 | 90/95(314) | Aryl-alcohol dehydrogenase-related oxidoreductase |  |
| C401_rfb25 | Leptospira borgpetersenii serovar Hardjo-bovis L550 | 94/98(282) | N-acetylneuraminic acid (sialic acid) synthetase | neuB |
| C401_rfb26 | Clostridium botulinum B1 str. okra | 24/51(290) | cytidyltransferase-like protein |  |
| C401_rfb27 | Leptospira borgpetersenii serovar Hardjo-bovis JB197 | 95/97(227) | hypothetical protein |  |
| C401_rfb28 | Leptospira borgpetersenii serovar Hardjo-bovis L550 | 97/98(401) | pyridoxal-phosphate-dependent aminotransferase |  |
| C401_rfb29 | Leptospira borgpetersenii serovar Hardjo-bovis L550 | 93/96(568) | ABC transporter permease/ATP-binding protein | sunT |
| C401_rfb30 | Leptospira borgpetersenii serovar Hardjo-bovis JB197 | 92/96(363) | hypothetical protein |  |
| C401_rfb31 | Leptospira borgpetersenii serovar Hardjo-bovis JB197 | 61/78(591) | hypothetical protein |  |
| C401_rfb32 | Leptospira interrogans serovar Copenhageni str. Fiocruz L1-130 | 71/87(217) | 6-phosphogluconolactonase/glucosamine-6-phosphate isomerase/deaminase |  |
| C401_rfb33 | Leptospira borgpetersenii serovar Hardjo-bovis JB197 | 74/85(275) | hypothetical protein |  |
| C401_rfb34 | Leptospira borgpetersenii serovar Hardjo-bovis L550 | 94/96(211) | phosphoheptose isomerase |  |
| C401_rfb35 | Leptospira interrogans serovar Lai str. 56601 | 99/99(251) | 2-dehydro-3-deoxyglucarate aldolase | kdsA |
| C401_rfb36 | Leptospira interrogans serovar Copenhageni str. Fiocruz L1-130 | 99/99(251) | 3-deoxy-manno-octulosonate cytidylyltransferase | kdsB |
| C401_rfb37 | Leptospira interrogans serovar Copenhageni str. Fiocruz L1-130 | 99/99(342) | oxidoreductase family protein |  |
| C401_rfb38 | Leptospira interrogans serovar Lai str. 56601 | 93/96(254) | inositol monophophatase family protein |  |
| C401_rfb39 | Thermosynechococcus elongatus BP-1 | 38/58(50) | bicarbonate transport system substrate-binding protein |  |
| C401_rfb40 | No homology gene was found | / | hypothetical protein |  |
| C401_rfb41 | Leptospira borgpetersenii serovar Hardjo-bovis JB197 | 85/89(47) | hypothetical protein |  |
| C401_rfb42 | Leptospira borgpetersenii serovar Hardjo-bovis JB197 | 92/96(79) | hypothetical protein |  |
| C401_rfb43 | Leptospira borgpetersenii serovar Hardjo-bovis JB197 | 85/92(221) | hypothetical protein |  |
| C401_rfb44 | Leptospira borgpetersenii serovar Hardjo-bovis JB197 | 89/93(349) | hypothetical protein |  |
| C401_rfb45 | Leptospira borgpetersenii serovar Hardjo-bovis JB197 | 94/98(318) | hypothetical protein |  |
| C401_rfb46 | Leptospira borgpetersenii serovar Hardjo-bovis JB197 | 93/95(320) | methylase/methyltransferase |  |
| C401_rfb47 | Leptospira borgpetersenii serovar Hardjo-bovis JB197 | 97/99(332) | dehydrogenase |  |
| C401_rfb48 | Leptospira borgpetersenii serovar Hardjo-bovis JB197 | 84/90(630) | hypothetical protein |  |
| C401_rfb49 | Leptospira borgpetersenii serovar Hardjo-bovis JB197 | 90/96(287) | glycosyltransferase |  |
| C401_rfb50 | Leptospira borgpetersenii serovar Hardjo-bovis L550 | 95/97(381) | PP-loop superfamily protein |  |
| C401_rfb51 | Leptospira borgpetersenii serovar Hardjo-bovis L550 | 86/96(204) | imidazole glycerol phosphate synthase subunit HisH |  |
| C401_rfb52 | Leptospira borgpetersenii serovar Hardjo-bovis L550 | 93/97(244) | imidazole glycerol phosphate synthase subunit HisF |  |
| C401_rfb53 | Leptospira borgpetersenii serovar Hardjo-bovis L550 | 91/96(390) | glycosyltransferase |  |
| C401_rfb54 | Leptospira borgpetersenii serovar Hardjo-bovis L550 | 92/96(332) | nucleoside-diphosphate-sugar epimerase |  |
| C401_rfb55 | Leptospira borgpetersenii serovar Hardjo-bovis L550 | 86/94(182) | nucleoside-diphosphate-sugar pyrophosphorylase |  |
| C401_rfb56 | Leptospira borgpetersenii serovar Hardjo-bovis L550 | 92/97(281) | glucose galactose epimerase | galE |
| C401_rfb57 | Leptospira interrogans serovar Lai str. 56601 | 88/95(344) | UDP-glucose 4-epimerase | galE |
| C401_rfb58 | Leptospira interrogans serovar Copenhageni str. Fiocruz L1-130 | 80/90(366) | nucleoside-diphosphate-sugar epimerase |  |
| C401_rfb59 | Leptospira interrogans serovar Copenhageni str. Fiocruz L1-130 | 80/90(376) | UDP-N-acetylglucosamine 2-epimerase | wecB |
| C401_rfb60 | Leptospira borgpetersenii serovar Hardjo-bovis L550 | 78/87(398) | glycosyltransferase |  |
| C401_rfb61 | Leptospira borgpetersenii serovar Hardjo-bovis L550 | 92/96(421) | undecaprenyl-galactosyl transferase | wcaJ |
| C401_rfb62 | Leptospira borgpetersenii serovar Hardjo-bovis JB197 | 92/95(423) | hypothetical protein |  |
| C401_rfb63 | Leptospira interrogans serovar Copenhageni str. Fiocruz L1-130 | 99/99(447) | polysaccharide biosynthesis export protein |  |
| C401_rfb64 | Leptospira borgpetersenii serovar Hardjo-bovis L550 | 86/94(300) | putative glycosyltransferase |  |
| C401_rfb65 | Leptospira interrogans serovar Lai str. 56601 | 99/99(297) | hypothetical protein |  |
| C401_rfb66 | Leptospira interrogans serovar Copenhageni str. Fiocruz L1-130 | 99/100(325) | glycosyltransferase |  |
| C401_rfb67 | Leptospira borgpetersenii serovar Hardjo-bovis L550 | 90/97(138) | sugar isomerase |  |
| C401_rfb68 | Leptospira borgpetersenii serovar Hardjo-bovis L550 | 86/95(363) | sugar pyridoxal-phosphate-dependent aminotransferase |  |
| C401_rfb69 | Leptospira borgpetersenii serovar Hardjo-bovis L550 | 94/97(241) | glycosyltransferase |  |
| C401_rfb70 | Leptospira borgpetersenii serovar Hardjo-bovis L550 | 93/96(281) | glycosyltransferase |  |
| C401_rfb71 | Leptospira interrogans serovar Copenhageni str. Fiocruz L1-130 | 93/95(574) | glycosyl transferase |  |
| C401_rfb72 | Leptospira interrogans serovar Lai str. 56601 | 99/99(186) | dTDP-4-dehydrorhamnose 3,5-epimerase | rmlC |
| C401_rfb73 | Leptospira interrogans serovar Lai str. 56601 | 97/99(306) | dTDP-4-dehydrorhamnose reductase | rmlD |
| C401_rfb74 | Leptospira interrogans serovar Copenhageni str. Fiocruz L1-130 | 99/100(336) | dTDP-glucose 4,6-dehydratase | rmlB |
| C401_rfb75 | Leptospira interrogans serovar Lai str. 56601 | 100/100(294) | glucose-1-phosphate thymidylyltransferase | rmlA |
| C401_rfb76 | Leptospira interrogans serovar Lai str. 56601 | 99/99(301) | glycosyl transferase |  |
| C401_rfb77 | Leptospira interrogans serovar Lai str. 56601 | 99/99(303) | dTDP-rhamnosyl transferase rfbF |  |
| C401_rfb78 | Leptospira interrogans serovar Lai str. 56601 | 98/99(282) | glycosyl transferase |  |
| C401_rfb79 | Leptospira interrogans serovar Copenhageni str. Fiocruz L1-130 | 99/100(464) | Sodium:sulfate symporter | citT |

*^a^* aa, amino acid.
